# Occurrence and seasonality of internal parasite infection in elephants, *Loxodonta africana*, in the Okavango Delta, Botswana

**DOI:** 10.1016/j.ijppaw.2015.01.004

**Published:** 2015-01-29

**Authors:** Lydia Baines, Eric R. Morgan, Mphoeng Ofthile, Kate Evans

**Affiliations:** a School of Biological Sciences, University of Bristol, Tyndall Avenue, Bristol, BS8 1TQ, United Kingdom; b Elephants for Africa, P.O. Box HA148 HAK, Maun, Botswana

**Keywords:** Nematode, Trematode, Coccidia, Elephant, Africa, Wetland, Transmission, Epidemiology

## Abstract

It is known from studies in a wide range of wild and domestic animals, including elephants, that parasites can affect growth, reproduction and health. A total of 458 faecal samples from wild elephants were analysed using a combination of flotation and sedimentation methods. Coccidian oocysts (prevalence 51%), and nematode (77%) and trematode (24%) eggs were found. Species were not identified, though trematode egg morphology was consistent with that of the intestinal fluke *Protofasciola robusta*. The following factors were found to have a significant effect on parasite infection: month, year, sex, age, and group size and composition. There was some evidence of peak transmission of coccidia and nematodes during the rainy season, confirmed for coccidia in a parallel study of seven sympatric domesticated elephants over a three month period. Nematode eggs were more common in larger groups and nematode egg counts were significantly higher in elephants living in maternal groups (mean 1116 eggs per gram, standard deviation, sd 685) than in all-male groups (529, sd 468). Fluke egg prevalence increased with increasing elephant age. Preservation of samples in formalin progressively decreased the probability of detecting all types of parasite over a storage time of 1–15 months. Possible reasons for associations between other factors and infection levels are discussed.

## Highlights

• The prevalence and density of internal parasite ova were recorded from wild elephants in the Okavango delta.
• Coccidian oocysts, and eggs of nematode and fluke parasites, were found to be common.
• Associations were found between infection and age, sex, group size composition, month and year of sampling.
• Coccidia appeared to be transmitted predominantly in the rainy and flood seasons.
• Formalin appeared to adversely affect recovery of all parasite taxa after prolonged storage.

## Graphical Abstract

**Figure:**
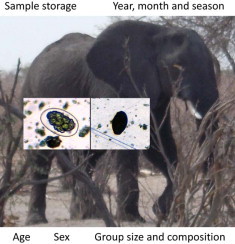

## 1 Introduction

Parasites can reduce body condition, reproductive success, and survival in their hosts (Irvine, 2006). Although parasite infections have been associated with mortality in the African elephant, *Loxodonta africana* (Vitovec et al., 1984; Obanda et al., 2011), research on the parasite fauna of this species is limited. More is known about parasites of Asian elephants (*Elephas maximus*), whose large captive population and significance to livelihoods underpin more detailed study (Lei et al., 2012). Apart from well recognised generalist taxa, most elephant-associated parasites so far described appear to be specific to either Asian or African elephants (Fowler and Mikota, 2006), suggesting that they have evolved to become host specific in the 7.6 million years since the African and Asian elephants diverged (Rohland et al., 2007). Due to the relatively limited amount of work that has been carried out on these parasites in African elephants, very little is known about their identity, occurrence, importance, life cycles and transmission dynamics.

Among African elephants, nematodes are frequently found (Kinsella et al., 2004; Fowler and Mikota, 2006; Thurber et al., 2011), with hookworms in particular reported to cause pathological lesions and haemorrhages in the bile ducts and liver, as well as the intestines (Obanda et al., 2011). The elephant-specific intestinal fluke *Protofasciola robusta*, likely to be an ancestral species within the Fasciolidae (Lotfy et al., 2008), has been associated with intestinal tissue damage, haemorrhage and death in free-ranging African elephants (Vitovec et al., 1984; Obanda et al., 2011). Coccidian infections, while apparently common, have not been widely associated with adverse clinical consequences (Fowler and Mikota, 2006).

This study sought to determine, by means of a coprological survey, the occurrence of and levels of infection with gastrointestinal parasites among African elephants in the Okavango Delta ecosystem, and to test for associations with potential drivers of transmission, including age, sex, group size and composition, season and year. Additionally, serial sampling of a small group of domesticated elephants at the study site was utilised to investigate the seasonality of transmission in this unusual and important part of the elephant's range.

## 2 Materials and methods

### 2.1 Study site

The study was conducted in the Ngamiland Wildlife Management Area 26 concession in the Okavango Delta, Botswana (19°25'N, 22°35'E). This is a private game concession of around 180,000 hectares, used for tourism, comprising riverine forest and grasslands, which flood seasonally along with the rest of the Delta. Rainfall is concentrated between November and February, and flood levels (from prior, upstream rains) rise from March to June, and then recede to September (Gumbricht et al., 2004). The study area has an estimated wild elephant population of 1350 (http://www.elephantdatabase.org/survey_aerial_total_count_strata/175, accessed 29th December 2014), as well as a small herd of domesticated African elephants used for transporting tourists on elephant-back safaris. Both populations have been subject to detailed behavioural studies in recent years (Evans and Harris, 2012; Evans et al., 2013), facilitating access to known groups and individuals for faecal sampling.

### 2.2 Faecal sampling

Fresh faecal samples were collected from individual free-living elephants during daylight hours (6 am–7 pm), between November 2008 and April 2012. Elephants were observed until they had defecated and had moved off to a safe distance. A sample was then taken, comprising separate aliquots from the surface and the interior of the dung bolus, to control for eggs having a heterogeneous distribution in the faeces. Only samples able to be collected within one hour of being dropped were taken. This was to avoid rapid parasite egg hatching or bolus drying, as well as disturbance and dispersion by insects such as dung beetles. Seven domesticated African elephants were kept at the study site. This group, known as the Abu herd, were used for elephant-back safaris, and enclosed at night but allowed to forage in the bush during daylight hours, as well as being walked regularly to water and on safari routes. This group therefore provided an opportunity to track temporal patterns in parasite load through longitudinal sampling, and hence reflect seasonal fluctuations in infection pressure to which wild elephants might also be exposed. Samples were collected from all seven members of the Abu herd over a shorter period (January to April, 2012), also during daylight hours. Samples were placed in plastic bags and stored in a cool box for transfer to the laboratory and processing on the day of collection.

The following information was collected for each sample: the date and time of collection, the age and sex of the elephant sampled, and the size and composition of its social group. Group composition was categorised as follows: Group 1 comprised either all females or females with males below the age of 15 years, while Group 2 comprised all males aged 15 years or more. These two categories represent the differential group living dynamics of wild African elephants. Female elephants remain in their matriarchal groups for life, while males are pushed out of these herds at the onset of puberty, and may remain solitary or form groups of their own (Evans and Harris, 2008, 2012). Elephants were assigned an age based on a number of visually observed variables, including body size, and tusk size and damage (Evans and Harris, 2008, 2012). The elephant population sampled has been the subject of long-term, on-going behavioural studies (Evans and Harris, 2012), and many of the observed elephants could be matched to a previously compiled identification database by observing ear markings, tusks and tail hair, and precise age consequently confirmed. This also minimised the risk of repeat-sampling of individuals.

For each sample collected between 12th November 2008 and 20th January 2012, three grams of faeces were weighed out, stored in a 15 ml storage pot and filled to the top with 10% formalin. These samples, hereafter referred to as formalin-preserved samples (FP-samples) were stored at ambient temperature and analysed between one and 15 months after collection. For samples collected between 21st January and 11th April 2012, hereafter referred to as unpreserved samples (UP-samples), three grams were also measured out, but were stored in a domestic refrigerator at around 4 °C, and analysed within 24 hours of collection.

### 2.3 Parasite enumeration

Nematode egg and coccidian oocyst density in faecal samples was estimated using a modified McMaster method (MAFF, 1986), with salt-sugar flotation solution (specific gravity 1.28) and a detection limit of 30 eggs per gram (epg). Briefly, 42 ml of water were added to each three gram sample, mixed thoroughly and then sieved. Two centrifuge tubes were filled with an aliquot of the sieved solution, and centrifuged for two minutes at 1500 rpm (400 *g*). The supernatant was then discarded and flotation solution added to the remaining sediment. The tubes were then inverted several times and a pipette was used to extract some of the mixed solution and place it in the chambers of a Fecpak slide (Fecpak Inc., New Zealand). This slide was used in preference to the standard McMaster slide because of the increased sensitivity, with one egg counted equating to 30 epg, compared with 50 epg using the standard modified McMaster method (Presland et al., 2005). The slides were left for two minutes to allow the eggs time to float to the surface before being examined under 10x objective (100 × total magnification) of a light transmission microscope. The prevalence of coccidian oocysts was recorded, and the number of nematode ova in each chamber was counted to estimate egg density.

Since some parasite ova, notably fluke eggs, could be too dense to float in salt-sugar solution, a sedimentation method (MAFF, 1986) was used to assess fluke prevalence. Faecal suspension was prepared as described in the flotation procedure above and topped up with water to 200 ml, mixed and poured into an inverse conical beaker. The beaker was left for three minutes to give fluke eggs time to sink. A pipette was then used to remove approximately 2 ml suspension from the very bottom of the beaker, and transfer it to the lid of a petri dish. After adding a drop of methylene blue stain, a graduated petri dish was then placed bottom-down on top of the lid to create an even layer of sediment, and the whole examined under 40x total magnification under a dissecting microscope. The number of fluke eggs seen was recorded. Early analysis of samples revealed that nematode eggs were frequently present in sediment fractions of FP-samples, while flotation tests on the same samples were negative for nematode eggs. Thereafter, nematode eggs were examined using both flotation and sedimentation methods. Sediment was examined in a petri-dish, as above, and the presence of fluke and nematode eggs recorded separately.

### 2.4 Statistical methods

Individual faecal samples were categorised by age, sex, month, season (wet, dry or flood), group size and group composition. Associations between these factors and the prevalence of coccidia and fluke, and of nematodes in FP-samples only, were investigated by binary logistic regression analysis, separately for each parasite type, in order to take account of potentially confounding interactions. All factors were included initially, and the least significant removed in turn until only significant predictors remained. The level of significance was set at p = 0.05. Logistic regression was not appropriate for nematode eggs in UP-samples, since observed prevalence was 100%. Instead, the effects of the same factors on nematode egg density were investigated by multiple linear regression analysis, following log_10_(x + 1) transformation to stabilise the variance. Because of apparently inconsistent flotation of nematode eggs preserved in formalin, nematode egg density was analysed only for UP-samples, and prevalence of the three parasite categories analysed for UP- and FP-samples separately. Nematode egg counts from samples analysed only using flotation, before the limitations of this method were known (see section 2.3 above), were discarded from the analysis of nematode egg prevalence. Trends in egg density in the captive Abu herd over the study period were assessed using two-tailed Pearson's correlation against time. One (3 month old) individual elephant was not found to be infected with any parasite at any time and was discarded from this analysis. Analyses were conducted using SPSS software (v16, SPSS Inc, USA).

## 3 Results

### 3.1 Sample size and distribution

A breakdown of samples collected by factor is given in Table 1. The median age of sampled elephants was 17 years (range 2–36), and the median group size 5 (range 1–85). A total of 61 UP-samples were analysed and 397 FP-samples, which were stored in formalin for between 1 and 15 months before analysis. A total of 197 samples were analysed before problems with nematode egg flotation were appreciated, and were excluded from analysis of nematode egg prevalence, leaving an effective sample size of 261 for this analysis. Sampling was skewed towards males early in the study to align with behavioural studies, and more females sampled later to achieve greater balance. In addition to samples from wild elephants, 79 faecal samples were collected from the seven individuals of the Abu herd.

### 3.2 Parasite prevalence and density in wild elephants

**Fig. 1:**
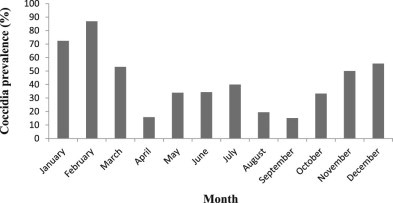
The prevalence of coccidial oocysts in FP-samples (formalin-preserved faecal samples) from wild elephants, in each month (2008 to 2012 combined).

Coccidian oocysts were recorded in 69% of UP-samples and 48% of FP-samples. For both sample types, prevalence varied seasonally (Fig. 1), and was significantly higher in January and/or February than in the reference month, March, which recorded intermediate prevalence (Tables 2 and 3). In FP-samples, coccidian oocysts were additionally more likely to be found in faecal samples taken in 2010 (prevalence 2008–12 = 47, 46, 65, 20, 56% respectively), and those that had been stored in formalin for less time (Table 3). Each additional month spent in formalin reduced the chance of finding coccidian oocysts using flotation by 13%. Although oocyst prevalence in males and females was very similar (48 and 49% respectively), when interaction with other factors was taken into account, oocysts were more likely to be found in samples from males than in those from females.

**Fig. 2:**
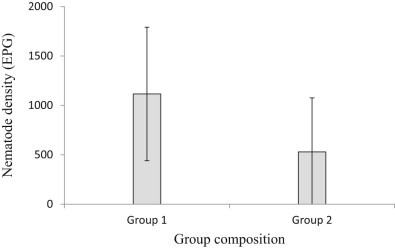
Nematode egg densities found in UP-samples (unpreserved, immediately analysed faecal samples) from wild elephants, categorised into two group types. Group 1 consists of groups with all female elephants and/or male elephants under the age of 15 years, and Group 2 consists of male elephants aged 15 years or more. Error bars show the standard deviation. EPG = eggs per gram of faeces.

**Fig. 3:**
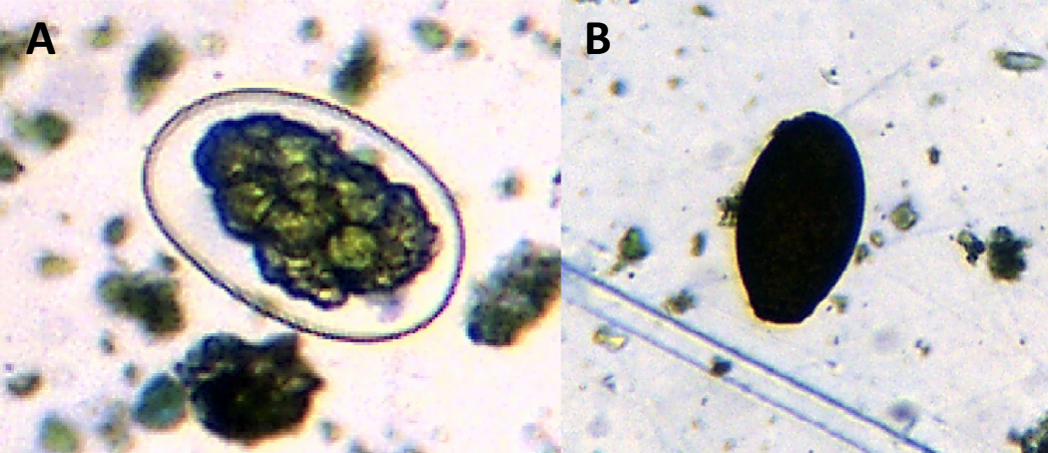
Photomicrographs of typical nematode (A) and trematode (= fluke, B) eggs found in elephant faecal samples. For dimensions see text.

Nematode eggs were present in 73% of FP-samples, and were more likely to be found in elephants from larger groups, and in samples that had been stored in formalin for less time (Table 4). For every additional elephant in a group, nematode eggs were 6.5% more likely to be found, and for every additional month spent preserved in formalin, they were 19% less likely to be found. Nematode eggs were present in all of the UP-samples analysed, rendering analysis of prevalence superfluous, but egg density varied. Samples from Group 1 (all female herds or those with males aged below 15 years) had significantly higher nematode egg density than those from Group 2 (males that are over the age of 15) (Table 5, Fig. 2). Nematode eggs were of typical strongyle-type morphology (Fig. 3), and of mean length (73 µm) (range 55–90) and width (46 µm) (range 35–55).

**Fig. 4:**
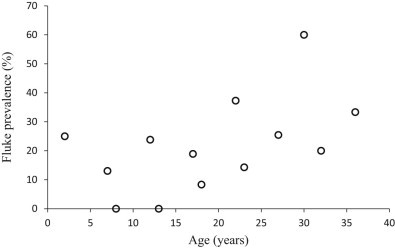
The prevalence of fluke (= trematode) eggs in wild elephants of different ages, using sedimentation of FP-samples (formalin-preserved faecal samples).

Fluke eggs were present in 26% of UP-samples. Prevalence was significantly higher in females (39%) than in males (10%) (Table 6). Fluke eggs were present in 23% of FP-samples, and were more likely to be found in samples collected in 2010 and 2011, and in those from older individuals (Fig. 4), and less likely to be found after longer storage in formalin (Table 7). Overall prevalence in years 2008–12 was, respectively, 12, 11, 27, 31 and 26%. Each additional month of storage in formalin decreased the chance of finding fluke eggs in an individual sample by 17%. Fluke eggs were operculate and measured 80–110 µm in length and 50–60 µm in width (Fig. 3), and were quite different in appearance to the classic *Fasciola*-type eggs seen in other large mammals in the study area.

### 3.2 Parasite occurrence in domesticated elephants

Samples were collected from the seven members of the Abu herd between January and April 2012. The group comprised six females aged 3 months to 37 years, and one male of 5 years. Over this period, a significant increase in nematode egg density was observed in two individuals (r = 0.727 and 0.759, n = 16 and 11, p = 0.001 and 0.008), with counts starting at 30 in both individuals and increasing steadily to 210 and 570 over the three month period. At the start of the study (21st January 2012), the six members of the Abu herd (excluding a new-born calf) were all infected with coccidia. However, from the 17th of March onwards, coccidian oocysts were no longer found in any of the samples collected. No fluke eggs were detected in the captive elephants at any time.

## 4 Discussion

This is, to our knowledge, the most extensive coprological parasite survey of wild elephants in Botswana to date. Specific identification of the parasite ova found was not possible, and would require corroborative *post mortem* recovery of adult parasites from elephants, which is rarely possible, given the high level of protection accorded to these animals and the rapid disintegration of carcasses. Advances in molecular methods provide opportunities for more specific studies in the future (McLean et al., 2012). Nevertheless, coprological surveys are useful to characterise broad patterns of infection at higher taxonomic levels (e.g. Thurber et al., 2011). In the present study, wild elephants in the Okavango Delta were found to be commonly infected with nematodes, coccidia and trematodes (=flukes). The morphology of the fluke eggs found was very similar to those of *Protofasciola robusta*, an intestinal fluke associated with emaciation and mortality in elephants in Kenya (Obanda et al., 2011). The seasonally wet conditions in the Okavango Delta probably provide suitable conditions for the life cycles of water-dependent fluke species. Lack of fluke infection in the domesticated elephants sampled, which have a more restricted range, suggests that infective stages might be distributed patchily in the environment. Coccidia and nematode ova were found in domesticated elephants as well as at high prevalence in wild elephants, demonstrating that conditions in the Delta are conducive to high levels of parasite transmission.

Considering fluke, nematode and coccidia ova together, year, month, sex, age, and group composition and size were all significantly associated with level of parasite infection.

Wild elephant samples collected in 2010 more commonly contained coccidian oocysts and fluke eggs than in other years, and fluke eggs were also more common than average in 2010 and 2011. Flood levels in the Delta were unusually high in 2010 (Tsheboeng et al., 2014), and this could have favoured parasite transmission through, variously and non-exclusively, more humid soil supporting parasite development and survival, better conditions for snail intermediate hosts of fluke, and higher host population density as a result of lower available land area. A more persistent effect might be expected for fluke than for intestinal coccidia, since flukes are generally longer-lived parasites.

Given the well-established links between climate and the transmission of many parasite taxa, it was surprising that no strong associations were found between season (rainy, flood and dry) and the prevalence or density of parasite stages in elephant faeces. However, transmission in one season can result in elevated parasite burdens in the next, given the time needed for parasite maturation, and prolonged parasite survival and propagule production. This would blur season–prevalence relationships. The prevalence of coccidia, but not of nematodes or fluke, was significantly associated with month. Oocysts were most likely to be observed in faecal samples in January and February, towards the end of the rainy season, after which prevalence declined. In the small number of serial sampled domesticated elephants, coccidian oocysts similarly disappeared from the faeces between February and March. These results suggest that the prevalence of coccidiosis is seasonal, and drops between the rainy season and the flood season. Other studies have shown high prevalence of coccidiosis in farmed livestock in the tropical rainy season (Rehman et al., 2011). Oocysts continued to be recorded in the present study at lower prevalence through the rest of the year. Parasite transmission could be enhanced by increased host density during the flood season, as elephants become concentrated on elevated land; however, there was no clearly increased prevalence at this time. In other systems in the region, e.g. antelopes in Zambia (Nalubamba et al., 2012), and in elephants in Nigeria (Mbaya et al., 2013), helminth prevalence typically peaks in the rainy season. The lack of a strong seasonal signal in helminth prevalence in the current study could be due to, among other factors, limited effects of climatic variation on transmission in this system, or parasite longevity damping fluctuations in transmission. In two of the longitudinally sampled domesticated elephants, nematode egg count increased substantially after the end of the rainy season, which would be consistent with infection from larvae that developed during the rains. However, further work is needed to characterise and explain the seasonal epidemiology of nematode infections in this system.

Male elephants were more likely to shed coccidian oocysts and less likely to shed fluke eggs than females, while nematode egg prevalence was unaffected by sex. Many mammal studies have found a male bias in parasitism, usually due to sexual dimorphism in behaviour or morphology, or by the effect of sex-specific hormones on the immune system (Zuk and McKean, 1996). If the latter effect is present in elephants, then bulls in musth, when plasma testosterone levels rise significantly (Ganswindt et al., 2010), might be expected to have increased parasite levels. However, too few musth bulls were encountered during the study to assess the effect of this heightened male hormonal state on parasite burden. A previous study in Namibia (Thurber et al., 2011) found that musth had no significant effect on parasite burden in bull elephants, suggesting that testosterone may not have a significant immunosuppressive effect in this species. Measurement of hormones in faeces may enable more detailed investigation of hormone–infection relationships in the future. Non-hormone related sexual dimorphism such as group structure, range and diet in male and female elephants may also contribute to the observed pattern of male biased coccidia infections.

Age was not associated with the prevalence of coccidia or nematode ova, nor nematode egg density. However, fluke prevalence increased with increasing elephant age. Flukes are typically long-lived within the final host, and this pattern is consistent with gradual accumulation of flukes through life, and limited host immunity. Unlike many livestock species (Armor, 1989), the elephants in this study do not appear to be acquiring immunity to parasites with age, or at least if such immunity occurs, it is not sufficiently strong to cause a detectable decrease in infection levels in previously exposed individuals. Similarly, a study on wild elephants in Namibia found that within family groups, nematode burden increased with age (Thurber et al., 2011), and this was attributed to older elephants eating more, and therefore being exposed to a greater number of parasites.

Elephant group size varied greatly in the stored sample study, ranging from two to 85 individuals, and the chance of nematode infection increased with increasing group size. A positive correlation between group size and parasite load in mammals was detected across species by meta-analysis (Cote and Poulin, 1994). The rate that the environment is contaminated by parasite eggs is positively correlated with the number of parasitised individuals in the population (Thurber et al., 2011). As larger herds have an increased probability of including infected individuals, it would be expected that larger group sizes lead to a high environment contamination rate, which in turn, leads to higher parasite levels. The host-density effect on parasite transmission may be exacerbated by the high water levels in the Delta, which force elephant group members to cluster together on dry ‘islands,’ thus increasing host density even further; although, there was no observed increase in infection levels during the flood season in the present study. Members of family groups (Group 1: females and males under the age of 15) had higher average nematode egg density than those in groups of mature males (Group 2). Thurber et al. (2011) also found that members of the matriarchal group had a higher nematode burden than solitary bull elephants. This might similarly be explained by higher contamination rates of frequented range areas by larger social groups. However, such processes might be expected to act across parasite taxa, and no relationship was found between group size and composition and the prevalence of coccidian or fluke ova.

Faecal egg count methodology was limited in this study due to the sinking in flotation solution of nematode eggs from elephant faecal samples after storage in formalin. This was unexpected but was overcome by changing the nematode detection method to include sedimentation as well as flotation, although this meant that only parasite prevalence, rather than density, could be estimated with confidence in stored samples. It was also found that increased time in formalin led to decreased detection of coccidia and fluke ova. It is possible that high ambient temperature adversely affects the integrity of parasite ova in faecal samples stored in formalin (Foreyt, 1986). This consideration should be borne in mind in other coprological studies of parasites in wildlife, in which prolonged storage of material is commonly used to overcome logistical barriers to immediate analysis.

## 5 Conclusions

Elephants in this study were found to commonly shed parasite ova in their faeces, including those of coccidia, nematodes and fluke. A wide range of factors was associated with parasite presence and density, including sex, age, group composition, group size, month and year. A significant effect of month on parasite prevalence was also found in sympatric domesticated elephants. In the case of coccidia, it appears that transmission is favoured in rainy and flood seasons. The high prevalence of fluke eggs is notable and could be due to the warm and wet conditions in the Okavango Delta. Further research is needed to establish whether internal parasites have any effect on individual fitness or population dynamics in this population, the extent to which transmission occurs between different sympatric host species, and to more fully understand the effects of climate and host biology in the epidemiology of parasite infections.

## Conflict of interest

The authors declare that there are no conflicts of interest in this revised article.

## Figures and Tables

**Table 1:** Number of samples taken from wild elephants, by categorical factor analysed. Total = 458. For distribution of samples along continuous variables, see text.

Factor	Level	Number of samples
Sex	Male	355
	Female	67
	Not known	36
Group composition	Group 1	187
	Group 2	241
	Not known	30
Season	Rainy	239
	Flood	182
	Dry	37
Year	2008	34
	2009	80
	2010	113
	2011	109
	2012	122
Month	January	32
	February	96
	March	45
	April	29
	May	53
	June	32
	July	15
	August	36
	September	33
	October	21
	November	30
	December	36

**Table 2:** Significant predictors of the prevalence of coccidial oocysts in UP-samples (un-preserved, or immediately analysed samples) from wild elephants, determined by binary logistic regression. The indicator month was March. Non-significant factors were age, sex, group size, group composition, season and year. B and Wald = test statistics, SE = standard error, df = degrees of freedom, OR = Odds Ratio.

	B	SE	Wald	df	p	OR	95% CI for OR	
							Lower	Upper
Month			8.303	3	0.040			
Month (February)	5.026	1.744	8.303	1	0.004	152.3	4.989	4649

**Table 3:** Significant predictors of the prevalence of coccidia in FP-samples (formalin-preserved samples) from wild elephants collected between 2008 and 2012 (n = 397), determined by binary logistic regression. The reference month was March, and the reference year 2009. Storage time was measured in months. Non-significant factors in the analysis were age, group size and group composition. B and Wald = test statistics, SE = standard error, df = degrees of freedom, OR = Odds Ratio.

	B	SE	Wald	df	p	OR	95% CI for OR	
							Lower	Upper
Sex			6.957	2	0.031			
Sex (male)	1.322	.607	4.741	1	0.029	3.75	1.14	12.4
Month			35.946	11	<0.001			
Month (Jan)	3.006	.871	11.914	1	0.001	20.2	3.67	111
Month (Feb)	4.343	.934	21.621	1	<0.001	76.9	12.3	479
Storage time	−0.135	.053	6.405	1	0.011	0.87	0.79	0.97
Year			43.882	4	<0.001			
Year (2010)	1.407	.399	12.458	1	<0.001	4.08	1.87	8.91
Year (2011)	−1.786	.506	12.444	1	<0.001	0.17	0.62	0.45
Year (2012)	−2.525	.910	7.702	1	0.006	0.08	0.01	0.48

**Table 4:** Significant predictors of the prevalence of nematode ova in FP-samples (formalin-preserved faecal samples) from wild elephants collected between 2008 and 2012. The least significant variables, month and year, were removed from the analysis. The remaining non-significant factors were age, sex and group composition. Storage time was measured in months. B and Wald = test statistics, SE = standard error, df = degrees of freedom, OR = Odds Ratio.

	B	SE	Wald	df	P	OR	95% CI for OR	
							Lower	Upper
Group size	0.065	.031	4.438	1	0.035	1.067	1.005	1.133
Storage time	−0.186	.065	8.261	1	0.004	0.831	0.732	0.943

**Table 5:** Significant predictors of nematode egg density in UP-samples (unpreserved, immediately analysed faecal samples) from wild elephants, using multiple linear regression on log-transformed egg counts. Non-significant factors were age, sex, group size, month, season and year. B = slope parameter, SE = standard error, t = test statistic, p = p-value, CI = confidence interval.

Model	Unstandardized coefficients		Standardized coefficients	t	p	95% CI for B	
	B	SE	Beta			Lower bound	Upper bound
Group composition	−.516	.178	−.595	−2.894	0.005	−.874	−.159

**Table 6:** Significant predictors of the prevalence of fluke eggs in UP-samples (unpreserved, immediately analysed faecal samples) from wild elephants, using binary logistic regression. The least significant factor, season, was removed from the analysis. The other non-significant factors were age, group size, group dynamic, month, season and year. B and Wald = test statistics, SE = standard error, df = degrees of freedom, OR = Odds Ratio.

	B	SE	Wald	df	p	OR	95% CI for OR	
							Lower	Upper
Male	−2.859	1.433	3.982	1	0.046	0.057	0.003	0.950

**Table 7:** Significant predictors of the prevalence of fluke eggs in FP-samples (formalin-preserved faecal samples) from wild elephants collected between 2008 and 2012 (n = 397), using binary logistic regression. The least significant variable, group composition, was removed from the analysis. The other non-significant factors in the analysis were sex, group size, month and season. The reference year was 2009. B and Wald = test statistics, SE = standard error, df = degrees of freedom, OR = Odds Ratio.

	B	SE	Wald	df	p	OR	95% CI for OR	
							Lower	Upper
Storage time	−0.171	.056	9.207	1	0.002	0.84	0.76	0.94
Year			15.220	4	0.004			
Year (2010)	1.626	.494	10.839	1	0.001	5.08	1.93	13.4
Year (2011)	1.513	.494	9.420	1	0.002	4.54	1.72	11.9
Age	0.039	.016	5.785	1	0.016	1.04	1.01	1.73
